# Regulation of Adipose Thermogenesis and its Critical Role in Glucose and Lipid Metabolism

**DOI:** 10.7150/ijbs.75488

**Published:** 2022-07-27

**Authors:** Linghui Wang, Yanhao Qiu, Hao Gu, Mailin Gan, Yan Zhu, Kangping Zhu, Lei Chen, Ye Zhao, Lili Niu, Shunhua Zhang, Xuewei Li, Li Zhu, Linyuan Shen

**Affiliations:** 1 Department of Animal Science, College of Animal Science and Technology, Sichuan Agricultural University, Chengdu 611130, China.; 2 Farm Animal Genetic Resource Exploration and Innovation Key Laboratory of Sichuan Province, Sichuan Agricultural University, Chengdu 611130, China.; 3 College of Life Science, China West Normal University, Nanchong 637009, Sichuan, PR China.; 4 Sichuan Dekon Livestock Foodstuff Group, Chengdu 610200, China.

**Keywords:** Brown adipose, Beige adipose, Coding RNA, Non-coding RNA, thermogenesis, Obesity, metabolic syndrome

## Abstract

The function of the adipose tissue is influenced by complex interactions between genetics, epigenetics, and the environment, and its dysfunction can cause a variety of metabolic diseases, such as obesity or type 2 diabetes (T2D). The beige/brown adipose tissue plays a crucial role in regulating glucose and lipid metabolism by increasing energy metabolism to generate heat. The adipose tissue thermogenic program is a complex network that involves many signaling pathways regulated by coding RNAs (cRNAs) that encode transcription factor, and non-coding RNAs (ncRNAs) including microRNAs (miRNAs) and long noncoding RNAs (lncRNAs). This article discusses factors that regulate adipose tissue thermogenesis, including cRNAs and ncRNAs, and the important role of thermogenic adipose tissue in obesity-related metabolic syndrome. Several studies have shown that some cRNAs and ncRNAs can modulate the thermogenic function of adipose tissue in different ways. This article reviews the roles of cRNAs and ncRNAs in regulating thermogenesis in the beige/brown adipose tissue and the important role of the beige/brown adipose tissue in maintaining the balance of glucose and lipid metabolism in the body.

## Introduction

With the improvement in quality of life, people's dietary patterns and structure have also changed. A large amount of high-fat and high-sugar diet culminates in excessive energy intake, and lack of exercise results in reduced energy consumption. The white adipose tissue (WAT) is the main source of energy and it stores excess energy in the form of triglycerides. However, the excessive deposition of WAT is the main cause of obesity syndrome 1. Today, obesity and its metabolic syndrome have become important factors that threaten human health, given that they are capable of increasing the risk of hypertension, coronary heart disease, chronic inflammation, T2D, and even the occurrence of cancer 2-6. Globally, obesity has nearly tripled since 1975, and as of 2019, 38.2 million children under five years of age were overweight or obese, according to the latest data from the World Health Organization (WHO). The problems of being overweight and obesity exist not only in high-income countries, but also in low- and middle-income countries, and are on the rise, especially in urban settings 7. Therefore, it is pertinent to explore ways to promote energy consumption and improve glucose and lipid metabolism in the body for the prevention or treatment of obesity.

The adipose tissue in mammals mainly includes the WAT, brown adipose tissue (BAT), and Beige adipose tissues in between. The adipose tissue plays a central role in regulating mammalian energy metabolism. The WAT is a major energy store in mammals, and when nutrient sources of energy are deficient, triglycerides in the WAT are broken down to produce fatty acids that are exported as fuel for other tissues, such as the muscle. Unlike the WAT, which stores energy, the BAT consumes energy through non-shudder thermogenesis that relies on uncoupling protein 1 (UCP1) to dissipate the proton gradient in the mitochondrial electron transport chain (ETC) to generate heat by oxidizing glucose and fatty acids to maintain the body temperature, a process called adaptive thermogenesis 8. In addition, the beige fat is an adipocyte intermediate between the WAT and BAT, and under certain conditions, such as cold exposure or induction by β3-adrenergic receptor (β3-AR) agonists, can stimulate the conversion of the WAT into Beige adipocytes with partial BAT features. Due to the correlation of functional properties with energy expenditure and obesity, BAT and beige adipocytes may be powerful therapeutic targets for obesity and its related metabolic diseases.

There are many kinds of RNA with complex functions in organisms. Generally, they are divided into two categories (depending on whether they encode proteins): cRNAs and ncRNAs. The former refers to messenger RNA (mRNA), while the latter includes many types, such as ribosomal RNA (rRNA), transfer RNA (tRNA), small nuclear RNA (snRNA), Piwi-interactingRNA (piRNA), miRNA, and lncRNA, among others. Among them, miRNAs and lncRNAs have received extensive attention in recent years. miRNAs are evolutionarily conserved 22-nucleotide (nt) single-stranded ncRNAs that act as post-transcriptional regulators by binding to the cis-elements in the 3'UTR region of mRNAs to play a major inhibitory role. In addition, studies have demonstrated that miRNAs can bind anywhere in the mRNA transcript to inhibit mRNA translation and affect mRNA stability 9. LncRNAs are a class of ncRNAs with a length of more than 200 nt that regulate gene expression at the chromatin, transcriptional, post-transcriptional levels, and binding to functional proteins or binding to chromosomes as precursors of miRNAs to regulate signaling pathways, etc. 10-14. The thermogenesis program of the adipose tissue is a very complex process, which involves a variety of genes and transcription factors that are closely related to thermogenesis, including UCP1, Prdm16, and PGC-1α, among others. Several studies on the regulation of adipose tissue thermogenesis by cRNA have confirmed that thermogenic genes and key factors of thermogenesis play a crucial role in these regulatory processes. For instance, FGF9 can increase adipose tissue heat production by regulating UCP1, thereby playing a role in regulating whole-body energy metabolism 15. There are similar studies on ncRNAs, such as miR-188, that can directly bind to the 3'UTR of Prdm16, a key transcription factor of the thermogenic program in the thermogenic adipose tissue, to inhibit the expression of Prdm16; Linc00473 can interact with mitochondrial and lipid droplet targets to regulate mitochondrial responses and lipolysis 16, 17. Therefore, in addition to cRNAs, miRNAs and LncRNAs may also play key roles in obesity and its related metabolic syndromes.

## The brown and beige adipose tissue characteristics of thermogenesis

Some features of beige adipocytes are similar to that of the BAT, such as having a large number of mitochondria, high expression of UCP1, and promoting energy consumption through non-shivering thermogenesis. However, there are some differences between beige and brown adipocytes, such as origin, ability to maintain thermogenic activity, thermogenic plasticity, and thermogenesis mechanisms. Beige adipocytes are derived from PDGFRα*^+^*, PDGFRβ*^+^*, SMA*^+^*, CD81*^+^*, and MYH11*^+^* progenitor cells. These pre-adipocytes give rise to beige adipocytes after cold exposure 18-21, while brown adipocytes are derived from MYF5*^+^*, PAX3*^+^*, and PAX7*^+^* smooth muscle cells and TRPV1*^+^* vascular smooth muscle cells 22, 23. The maintenance of beige fat requires the inhibition of autophagy and is closely related to the number of mitochondria. When external stimuli, such as cold exposure, are removed, the beige fat in mice loses its original morphological and molecular characteristics, and the expression level of UCP1 decreases significantly, turning into an adipocyte with high expression of white adipocyte marker genes. On the other hand, brown adipocytes can maintain a higher thermogenic activity 24-27. The thermogenic activity of brown adipocytes can be increased to a certain extent under external stimuli, while the expression of UCP1 in beige adipocytes is not as high as that in the BAT; however, the expression of UCP1 can be strongly induced by external stimuli, thus showing greater thermogenesis plasticity 28, 29.

**Figure 1:**
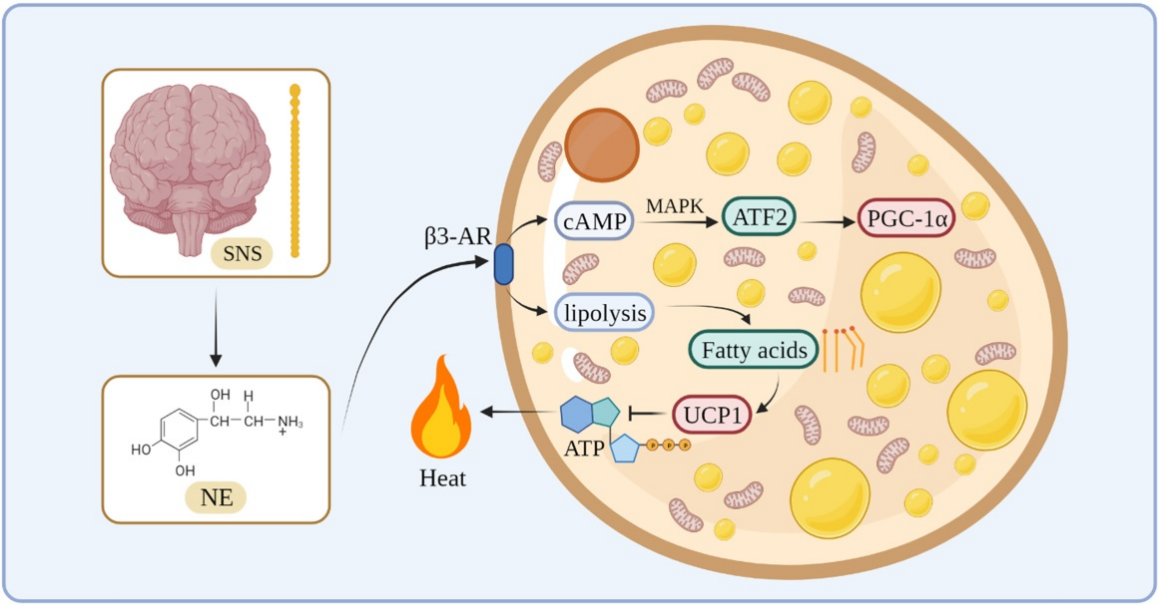
The thermogenesis mechanism of BAT.

In terms of the thermogenesis mechanism, the classical BAT mainly stimulates β3-adrenergic receptors to increase intracellular lipolysis through norepinephrine (NE) released by the sympathetic nervous system. Fatty acids are used for oxidation and they bind directly to activate UCP1, which uncouples oxidative phosphorylation and mitochondrial ATP synthesis to generate heat 30-32. During this process, NE activates cyclic adenosine monophosphate (cAMP) through β3-AR on the cell membrane of the adipose tissue, and cAMP stimulates cAMP-dependent protein kinase A to phosphorylate downstream proteins, such as cAMP-responsive element-binding protein (CREB) 33. At the same time, cAMP activates transcription factor 2 through the p38/mitogen-activated protein kinase pathway, thereby increasing the expression of the downstream transcription factor PGC-1α 34, 35 **(Figure 1)**.

The thermogenesis of beige adipocytes is closely related to PGC1-α, which can stimulate the expression of UCP1 and control mitochondrial biogenesis and oxidative metabolism 36. In addition, the β3-adrenergic signaling pathway and Prdm16 are involved in the process of white fat browning. For instance, Prdm16 plays an important role in the browning of the WAT, which is induced by PPAR-γ agonists. Prdm16 can combine with PPAR-γ to form a transcription complex to promote the expression of downstream genes, such as UCP1, and promote the transformation of the WAT into beige fat 37. Recent studies have identified mechanisms by which the beige fat regulates systemic metabolism, such as creatine cycling and Serca2b-mediated calcium cycling 38, 39. There are several studies on humans showing that cold exposure recruits brown and beige adipocytes, leading to increased energy expenditure and beneficial effects on glucose metabolism 40, 41. Due to this thermogenic plasticity and association with obesity in humans, thermogenic adipocytes represent an attractive therapeutic target for obesity and its related metabolic diseases.

## Regulation of adipose tissue thermogenesis via regulation of key thermogenesis factors

Several studies have confirmed that there are many factors in the adipose tissue that can affect adipose tissue thermogenesis, such as UCP1, Prdm16, and PGC-1α, which play an important role in regulating the process of adipose tissue thermogenesis. UCP1 is a mitochondrial transmembrane protein that uncouples oxidative phosphorylation of the inner mitochondrial membrane from ATP generation, allowing protons accumulated in the mitochondrial intermembrane space to return to the matrix side to support the robust thermogenic capacity of the BAT. Notably, brown adipocytes lacking UCP1 exhibited respiration rates comparable to those of wild-type cells under unstimulated conditions. Similarly, under unstimulated conditions, animals with structural inactivation of the UCP1 gene also exhibited comparable respiration rates to those of wild-type animals. This further illustrates that UCP1 plays a crucial role in thermogenesis 42. Therefore, UCP1 dependence is not the only pathway for shiver-independent thermogenesis. Also, UCP1-deficient animals can adapt to cold environments by enhancing compensatory mechanisms for UCP1-independent non-shiver thermogenesis. Prdm16 is an important transcription factor in the differentiation of brown adipocytes, which plays an important role in maintaining the special morphological characteristics and cell function of brown adipocytes. In addition, Prdm16 can specifically activate adipogenesis in brown adipocytes; it also forms a transcriptional complex with C/EBPβ to control the cell fate switch in brown adipocytes 43. PGC-1α is a transcriptional coactivator that mediates many biological programs related to energy metabolism and is also a central regulator of brown adipose thermogenesis. It is highly expressed in the BAT and it regulates the expression of UCP1 and thermogenesis in the BAT. Therefore, many cRNAs and ncRNAs that affect adipose tissue thermogenesis are inevitably involved in the regulation of these important thermogenesis factors.

### cRNAs regulates transcription factors of genes associated with thermogenesis

**Figure 2:**
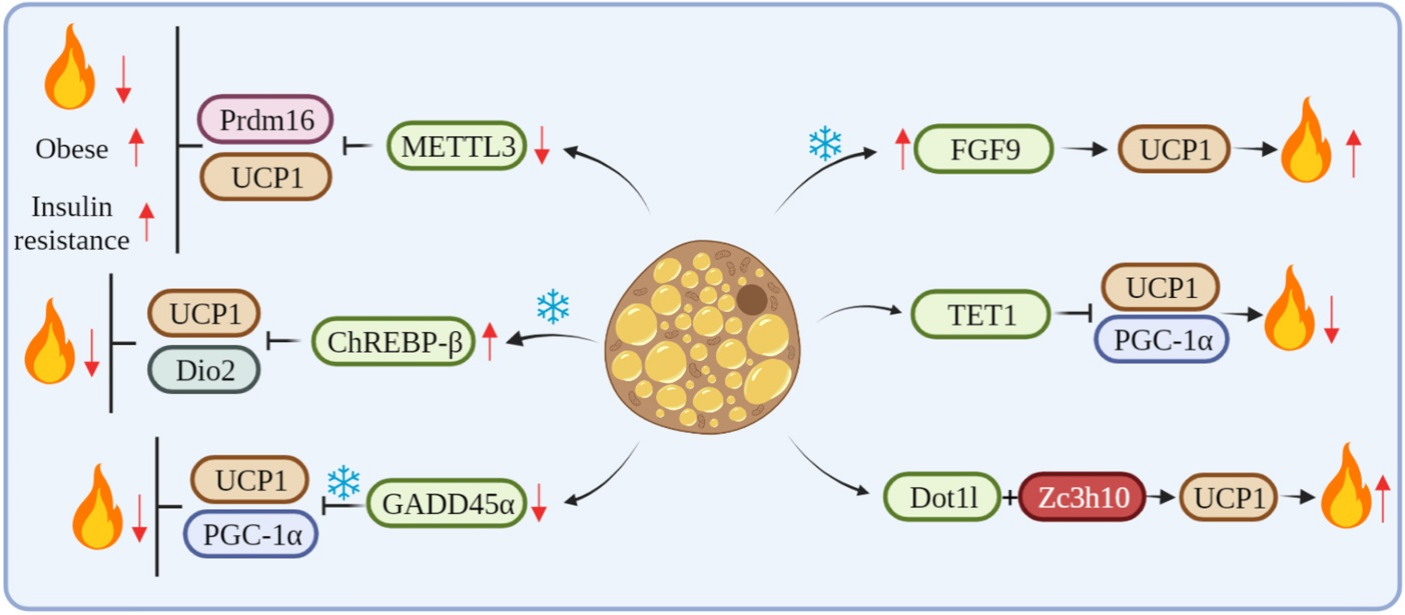
cRNAs regulate adipose tissue thermogenesis by affecting key thermogenesis factors.

METTL3, a key RNA methyltransferase, is highly expressed in the interscapular BAT and plays a critical role in its development and maturation. Conversely, the specific deletion of METTL3 in the BAT reduces the expression of Prdm16 and UCP1 transcripts, which impairs BAT maturation, thus resulting in a marked reduction in BAT-mediated adaptive thermogenesis and high-fat diet (HFD)-induced obesity and systemic insulin resistance 44. Klf9 regulates energy metabolism by stimulating the expression of PGC-1α. Klf9-deficient mice have abnormal BAT and a large accumulation of lipid droplets, resulting in impaired thermogenesis in mice 45. The mRNA level of ChREBP-β was increased after cold exposure. The overexpression of ChREBP-β resulted in an increase in lipid droplet size and a decrease in mitochondrial content in mouse BAT at room temperature. Also, the overexpression of ChREBP-β down-regulated mitochondrial biogenesis and the expression of autophagy and respiration-related genes. In addition, the overexpression of ChREBP-β significantly suppressed the expression of thermogenic genes, including UCP1 and Dio2, thus identifying ChREBP-β as a negative regulator of thermogenesis in brown adipocytes 46. GADD45α mRNA expression is associated with obesity and may regulate lipid metabolism and brown adipogenesis. GADD45α knockout significantly upregulated the expression of Ki67 and other cell cycle markers and promoted the proliferation of brown adipocytes. Knockout mice of GADD45α have impaired UCP1 function and thermogenic response in cold. Also, GADD45α deletion promotes mitochondrial biogenesis through both PGC1-α and UCP1 mRNA and protein expression *in vitro* and *in vivo* 47. FGF6/9 expression in mouse adipose tissue is upregulated by exercise and cold, whereas FGF9 expression in human neck fat is significantly correlated with UCP1 expression. The loss of FGF9 impairs BAT thermogenesis, while the *in vivo* overexpression of FGF9 increases UCP1 expression and the thermogenic capacity of the adipose tissue, FGF9 can increase adipose tissue thermogenesis by regulating UCP1*,* thus regulating whole-body energy metabolism 15. TET1 acts as a suppressor of key thermogenic genes, such as UCP1 and *Ppargc1α*, in beige adipocytes. The adipose tissue-selective knockout of TET1 can improve cold tolerance, increase energy expenditure, and prevent diet-induced obesity and insulin resistance in mice 48. Dot1l can interact directly with Zc3h10, which recruits Dot1l to the promoter regions of thermogenic genes, such as UCP1, thereby activating thermogenic gene programs. The deletion of Dot1l can prevent the activation of UCP1 and other thermogenic genes, thereby reducing thermogenic capacity and energy expenditure and promoting obesity 49 **(Figure 2)**.

### ncRNAs directly regulate genes associated with thermogenesis

In terms of adipose tissue thermogenesis, UCP1 has become an important indicator for evaluating thermogenesis capacity. According to the expression changes of UCP1, it is often possible to judge whether various factors have a positive or negative effect on adipose tissue thermogenesis. In brown adipocytes, the expression level of miR-23b-5p gradually decreased with the differentiation of the mouse and human brown adipocytes. The overexpression of miR-23b-5p resulted in the decrease of the mRNA and protein levels of the brown adipocyte marker gene UCP1. It is suggested that miR-23b-5p may be a negative regulator of the thermogenic gene program in brown adipocytes 50. Some researchers screened some differentially expressed miRNAs by small RNA sequencing of goat BAT and WAT and found that the overexpression of miR-433 reduced lipid accumulation in brown adipocytes and significantly reduced the expression of UCP1. Similarly, the overexpression of miR-433 significantly reduced the UCP1-dependent oxygen consumption rate of brown adipocytes, thus suggesting that miR-433 plays a repressive role in the differentiation and thermogenesis of goat brown adipocytes 51. In addition, there are human-related studies demonstrating that ncRNAs can affect the expression of UCP1. The researchers found that the expression level of miR-27b-3p in the visceral adipose tissue (VAT) of obese subjects was much higher than that of normal-weight subjects. At the same time, the expression of miR-27b-3p in the VAT was negatively correlated with UCP1 52.

By far, the most potent and best-performing interventions for inducing energy expenditure in rodents and humans are adrenergic pathway stimulators and peroxisome proliferator-activated receptor agonists, which are molecules that trigger the promotion of multiple pathways of thermogenic gene expression. Most of these molecules depend on the activation of the transcriptional co-activator PGC-1α 53-56. PGC-1α is a major regulator of UCP1-mediated BAT thermogenesis and an effective target of miR-122; however, the specific underlying mechanism is yet to be explored 57. Studies have shown that the expression of miR-494-3p in primary beige adipocytes of mice is decreased and that the protein levels of PGC-1α and UCP1 are up-regulated after cold stimulation and β3-adrenergic stimulation. In addition, it is confirmed that miR-494-3p can directly target PGC-1α to regulate thermogenesis in beige adipocytes 36. The miR-199a/214 cluster directly targets PGC-1α to suppress brown adipocyte differentiation and beige adipocyte development, thereby suppressing the expression of thermogenic genes, which indicates that the miR-199a/214 cluster is a key negative factor for brown and beige adipocyte development and thermogenesis regulation 58. The knockdown of LncRNA H19 in brown adipocytes resulted in the reduced expression of PGC-1α and other markers of brown adipogenesis, while its overexpression supports oxidative metabolism, uncoupled respiration, and extracellular acidification in differentiated brown adipocytes rate and makes brown adipocytes more sensitive to stimulation by β3-adrenoceptor agonists 59. The overexpression of LncRNA 2310069B03Rik reduced PGC-1α levels, concomitantly downregulated expressions of Dio2, FGF21, and Prdm16, and reduced the thermogenic capacity of thermogenic adipose tissues 60.

Prdm16 is a key cell-autonomous regulator. Complex interactions between *Prdm16* and other factors are required to control the differentiation of adipocytes into thermogenic adipose tissues. Therefore, Prdm16 is required for the thermogenic adipose tissue to exert its biological functions. In addition, Prdm16 is also a downstream effector of many miRNAs/lncRNAs. For example, miR-188 directly binds to the 3'UTR of Prdm16 mRNA, inhibits the expression of Prdm16 in the adipose tissue of aging mice, and regulates aging-related metabolic phenotypes in a way that affects thermogenesis 17. A study screened and isolated miR-191-5p, which plays an important role in the control of the WAT browning, and confirmed that the up-regulation of miR-191-5p effectively increased the expression of Prdm16 and promoted the browning of the WAT 61. The miR-199a/214 cluster expression was decreased during brown and beige adipocyte differentiation and in response to cold exposure or β-adrenergic receptor activation, which suggests that the miR-199a/214 cluster plays a negative role in adipose tissue thermogenesis. Our results confirmed that the miR-199a/214 cluster was able to directly target Prdm16 to suppress brown adipocyte differentiation and beige fat development 58. In addition to the miRNA targeting Prdm16 to regulate adipose tissue thermogenesis, Prdm16 can also regulate the levels of certain miRNAs. For example, Prdm16 can up-regulate miR-193b-365 through PPARα. miR-193b-365 is an important regulator of BAT differentiation and can induce myoblasts to differentiate into brown adipocytes 62. After cold exposure, miR-27 was downregulated during brown adipogenesis in the BAT and primary preadipocytes *in vitro* and affected brown adipose tissue thermogenesis by directly targeting and negatively regulating the key factors of brown adipose tissue thermogenesis Prdm16 and PPARα 63. In addition, some lncRNAs are also able to regulate the expression level of Prdm16 in the adipose tissue, as identified and functionally verified by Chunming Ding^64^. The loss of function of lnc-dPRDM16 not only downregulates the expression of Prdm16 but also significantly inhibits browning in cell cultures undergoing adipogenesis, which suggests that lnc-dPRDM16 is an important regulator of brown fat development 64. The up-regulation of LncRNA ROR increases the mRNA and protein expression levels of Prdm16 and promotes the differentiation of human adipose stem cells into brown adipocytes 65.

**Figure 3:**
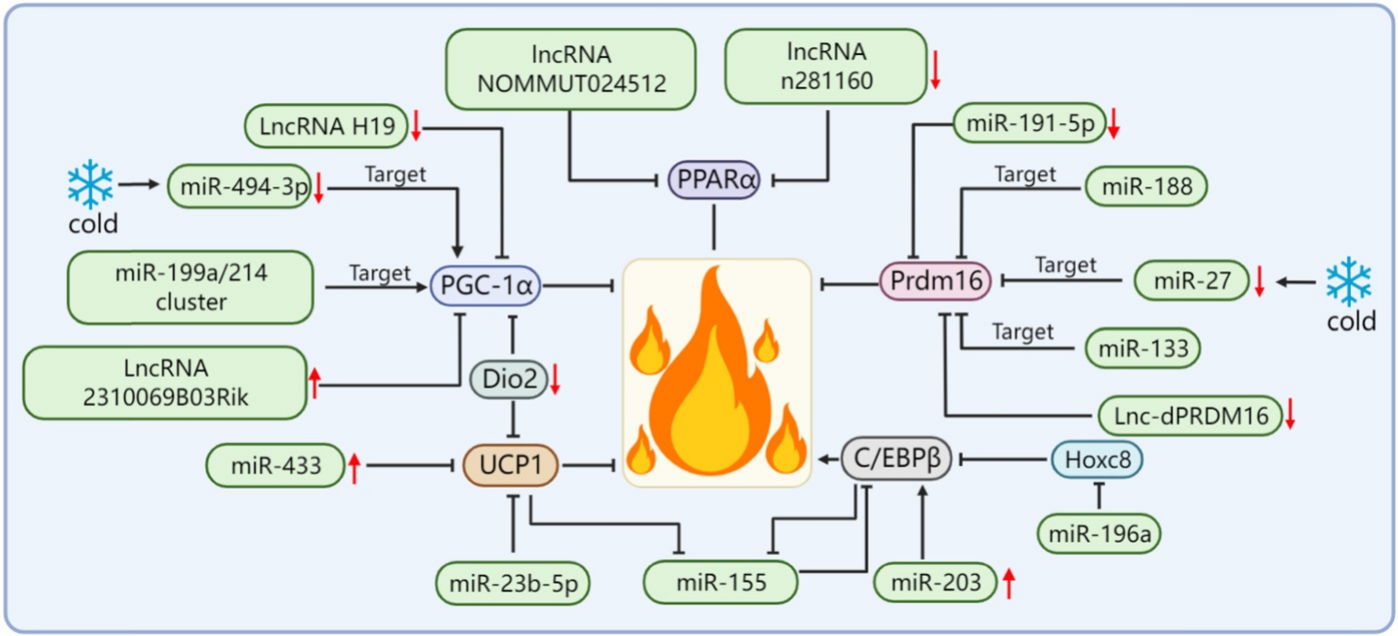
miRNA/lncRNA regulates adipose tissue thermogenesis by affecting key thermogenesis factors.

In addition to Prdm16 and PGC-1α, miRNA/lncRNA can also affect the thermogenic function of thermogenic adipose tissues by regulating other thermogenesis-related genes, such as C/EBPβ, Dio2, and PPARα. In addition to its role in adipogenesis, C/EBPβ is required for BAT development and can synergize with Prdm16 as a key switch in brown adipocyte development. In addition, C/EBPβ is also a key transcriptional regulator of UCP1 expression and the thermogenic program-inducing factor 66-68. Yong Chen *et al.* found that there is a mutual negative regulation between miR-155 and C/EBPβ and that miR-155 transgenic mice showed decreased BAT mass and UCP1 expression, which led to a significant reduction in BAT thermogenesis 69. The study found that the expression of miR-203 was positively correlated with energy expenditure. The overexpression of miR-203 could promote the WAT browning in cold-exposed mice, as activated by up-regulation of cAMP-dependent C/EBPβ, and improve glucose tolerance in HFD-fed mice by inhibiting the IFN-γ signaling pathway 70. The target gene of miR-196a is Hoxc8, and Hoxc8 directly targets C/EBPβ. miR-196a inhibits C/EBPβ by inhibiting *Hoxc8*, thereby activating the brown fat thermogenic program 71. Dio2 plays an important role in enhancing adrenergic signaling and promoting the induction of UCP1 and PGC1-α in brown adipocytes. Dio2-null mice showed impaired adaptive thermogenesis 72, 73. Whereas miR-382 directly targets Dio2, the down-regulation of miR-382 leads to increased Dio2 expression, thereby increasing fat browning and energy expenditure 74. PPARα is a well-known important thermogenesis regulator of brown fat. A found that the expression of two lncRNAs (NOMMUT024512 and n281160) in the BAT of aged mice was significantly down-regulated. Interestingly, their adjacent coding gene was PPARα. The knocking out of these two lncRNAs resulted in the decreased expression of PPARα; however, the expression of other nearby or upstream genes was not affected. Thus, these two lncRNAs may be involved in the thermogenic function of BAT by acting on PPARα 75 **(Figure 3)**.

In addition, some non-canonical factors can also be involved in the activation or inhibition of the thermogenic program as targets of miRNA/lncRNA to regulate the thermogenic function of the thermogenic adipose tissue (see **Table 1** for details).

## Regulates adipose tissue thermogenesis and energy metabolism by affecting mitochondrial biosynthesis and mitochondrial function

It is known that the rapid activation of BAT-mediated non-shivering thermogenesis is due to the proximity of lipid droplets within brown adipocytes to mitochondria and these triglycerides are released upon adrenergic stimulation to drive rapid mitochondrial oxidation 81, 82. Therefore, the number and function of mitochondria are closely related to the normal thermogenic function of the thermogenic adipose tissue, and the functional impairment of mitochondria may lead to a decrease in the thermogenic capacity of the thermogenic adipose tissue.

Mitochondria are important for brown fat energy dissipation and many RNAs can regulate adipose tissue thermogenesis by affecting mitochondrial biogenesis and function. Mutations in Opa1, a member of the mitochondrial plastic protein family, cause severe mitochondrial dysfunction. Studies have shown that the overexpression of Opa1 in mice favors the expansion and browning of the WAT, thereby improving glucose tolerance and insulin sensitivity 83. Bola3 is among the 17 mitochondrial Fe-S cluster assembly genes. The downregulation of Bola3 decreased the expression of mitochondrial-related genes and respiratory chain complexes, decreased mitochondrial formation, and suppressed isoproterenol-stimulated lipolysis, thereby inhibiting the thermogenic activity of beige adipocytes 84. LETM1 is a mitochondrial outer membrane protein and LETM1 knockout mice showed brown adipocytes with reduced mitochondrial abundance and concomitant reductions in mitochondrial DNA copy number and thermogenic gene expression in the BAT. Also, these mice were unable to maintain body temperature under cold exposure conditions without food. The expression of LETM1 is essential for mitochondrial structure and function and thermogenesis in brown adipocytes 85. Proper mitochondrial dynamics are critical for adipocyte thermogenesis and it was found that the mitochondrial fission protein dynamin-related protein 1 (DRP1) is highly expressed in the BAT and increases with the differentiation of the brown adipocyte. The inhibition of DRP1 expression attenuates mitochondrial biogenesis in beige adipocytes, which confirms that DRP1 plays an important role in beige and brown adipogenesis and thermogenesis 86.

In addition, many ncRNAs play crucial roles in regulating mitochondrial biogenesis and function in adipose tissue. miR-494-3p acts as a regulator of mitochondrial biogenesis, targeting the mitochondrial marker genes Tfam, FoxJ3, and CREB1 to reduce mitochondrial biogenesis in adipocytes 36. High expression of miR-204-5p impairs mitogenesis in adipose tissue 87. β3-adrenergic agonists can induce an increase in mitochondrial production, whereas miR-125b-5p mimic treatment impaired β3-adrenergic agonist-induced mitochondriogenesis, inhibited citrate synthase mRNA expression, and reduced the expression levels of beige adipocyte markers, such as UCP1 and Cpt1m 88. The overexpression or downregulation of miR-199a-3p significantly affected the expression of mt-ND1 and mt-CYTOB, thus suggesting a role for miR-199a-3p in regulating mitochondrial content 89. The mammalian target of rapamycin (mTOR) is a 250 kDa conserved Ser/Thr kinase. The mTOR signaling pathway is a central regulator of cellular energy metabolism and plays an important role in maintaining BAT thermogenesis 90-92. Studies have shown that miR-199a-3p is down-regulated during thermogenesis in mouse brown adipocytes, whereas the overexpression and down-regulation of miR-199a-3p in brown adipocytes *in vivo* decreased and increased mTOR mRNA and protein levels, respectively. In addition, the overexpression of miR-199a-3p impairs the metabolic characteristics of brown adipocytes, such as a reduction in mitochondrial DNA content and mitochondrial respiration, with some impact on brown adipogenesis and thermogenesis 89.

LncRNA AK079912 is a lncRNA enriched in brown adipocytes; its expression was increased in brown preadipocyte differentiation and white adipocyte browning after cold stimulation. The ectopic expression of LncRNA AK079912 in white preadipocytes was upregulated with the expression of thermogenesis-related genes. Mechanistically, the inhibition of lncRNA AK079912 resulted in decreased mitochondrial copy number and protein levels of ETC complexes, thereby affecting the thermogenic capacity of the thermogenic adipose tissue 80. In addition, lncRNA GM13133 is also a lncRNA specifically expressed in the BAT and actively mediated by cold exposure, β3-adrenergic agonist, and cAMP stimulation. A forced expression of lncRNA GM13133 can enhance mitochondrial biogenesis in adipocytes and induce the expression of brown adipocyte-specific markers 93. The deletion of lncRNA Ctcfls in beige adipocytes resulted in reduced mitochondrial biogenesis, impaired mitochondrial oxidative capacity, and severe disturbance of the thermogenic potential of beige adipocytes, thus suggesting that lncRNA Ctcfls play an important role in the thermogenic program in the thermogenic adipose tissue and is required in the maintenance of the thermogenic phenotype of mature cells 94. In addition, lncRNA FOXC2-AS1 can promote the increase of mitochondrial content by inhibiting the autophagy signaling pathway, thereby promoting the transformation of adipocytes from white to brown 95. Autophagy is very important for the maintenance of thermogenic adipocytes; it causes an increase in mitochondrial content and maintains thermogenesis by inhibiting mitochondrial clearance 25, 96.

**Figure 4:**
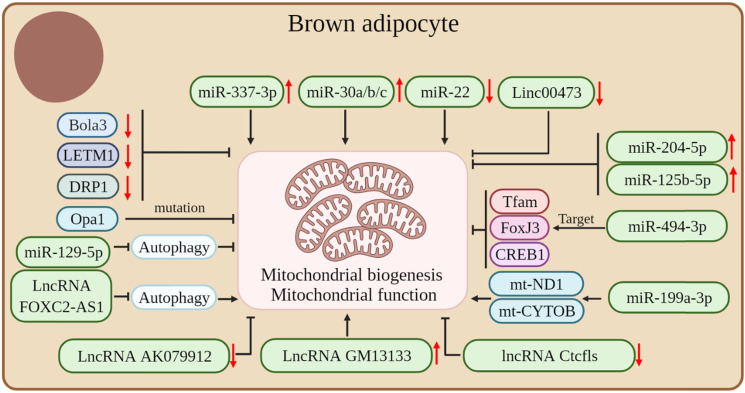
Coding and non-coding RNAs regulate mitochondrial biogenesis and mitochondrial function in thermogenic adipocytes.

In addition, studies have demonstrated that some ncRNAs can affect the thermogenic capacity of the adipose tissue by affecting mitochondrial function in the adipose tissue. For instance, miR-129-5P may disrupt endoplasmic reticulum homeostasis by blocking autophagy, thereby reducing mitochondrial function in beige adipocytes and BAT 97. The expression level of miR-337-3p in the BAT was significantly higher than that in the WAT. Also, the overexpression of miR-337-3p in brown preadipocytes resulted in the increased expression of mitochondrial markers, thus suggesting that miR-337-3p can enhance adipocyte browning 77. MiR-30a is enriched in the BAT and the forced expression of miR-30a increases mitochondrial volume and mitochondrial respiration in human white adipocytes, making them characteristic of brown adipocytes 98. The forced expression of miR-30b/c was able to significantly increase mitochondrial respiration in primary adipocytes from the subcutaneous WAT, suggesting that miR-30b/c promotes the development of beige fat 99. The level of miR-22 is gradually increased during the differentiation of white, beige, and brown adipocytes *in vitro*, whereas the deletion of miR-22 protects HFD-induced mice from mitochondrial dysfunction in the WAT and BAT and induces WAT browning 57. The miRNA Let-7i-5p can target genes involved in mitochondrial function and impair mitochondrial oxygen consumption, thereby affecting the function of beige adipocytes 100. Decreased levels of Linc00473 lead to a significant decrease in mitochondrial respiration rate in the adipose tissue and vice versa, which suggests an interaction between Linc00473 and mitochondrial respiration and that Linc00473 may be a key regulator of thermogenic adipocyte function in humans 16 **(Figure 4)**.

## Effects of the thermogenic adipose tissue on obesity-related metabolic diseases

Obesity occurs when energy intake and expenditure are chronically unbalanced; it also increases the risk of cardiovascular disease (CVD), non-alcoholic fatty liver disease (NAFLD), T2D, and other diseases. At present, there are two ways to treat obesity: reduction of energy intake and increase in energy consumption. As studies on brown and beige adipose tissue increase, more attention is paid to the function of the thermogenic adipose tissue to increase mitochondrial respiration and energy metabolism. In animal models, the large increase in the amount and activity of brown and beige fat resulted in a dramatic improvement in metabolism, while no pathological changes were detected. This suggests that thermogenic adipose tissue can regulate the body's energy metabolism safely.

### Effects of the thermogenic adipose tissue on NAFLD

In recent years, non-alcoholic fatty liver disease has become an important health problem related to obesity. A considerable number of patients with NAFLD will develop liver cirrhosis and liver tumors 101. The vast majority of NAFLD occurs in people with obesity and related metabolic abnormalities, such as diabetes and dyslipidemia 102, 103. There is an urgent need to find a means of treating or delaying the progression of NAFLD. A chronic systemic inflammatory state, one of the hallmarks of obesity, may contribute to the pathology of NAFLD and T2D. Also, combating systemic inflammation and consequent liver fibrosis may alleviate this pathological state. It has been shown that the depletion of UCP1 triggers severe inflammation and pathological conditions in the liver but not obesity and changes in systemic energy expenditure. Hepatic stellate cells (HSCs) are the major cell types that drive tissue fibrosis and inflammation. Moreover, it is found that the livers of UCP1-deficient mice show an increased abundance of proteins associated with HSCs activation. In addition, the regulation of neutrophil-mediated immunity and proinflammatory cell factor production of proteins was also significantly increased, which is strongly associated with nonalcoholic steatohepatitis (NASH) and insulin resistance. Furthermore, UCP1-deficient mice had significantly elevated circulating alanine aminotransferase and aspartate aminotransferase levels, which are hallmarks of liver damage. Thus, this study demonstrates that the content and activity of UCP1-positive adipocytes determine the ability of the BAT to clear and break down circulating succinate and that UCP1-positive adipocytes regulate HSCs and macrophage populations in the liver by preventing SUCNR1 agonists from, thereby strongly antagonizing liver inflammation and pathology 104. In addition, in a study investigating whether increasing non-shivering thermogenesis could improve pre-existing NASH in mice, β3-AR agonist treatment restored the BAT function, reduced body weight, improved glucose tolerance, and decreased the liver lipid content of HFD-induced mice; however, the activity scores of liver inflammation and NAFLD were not affected. However, β3-AR agonists enhanced weight loss and glucose tolerance, improved hepatic steatosis and hepatocyte damage, and further reduced hepatic inflammation in mice with caloric restriction 105.

### Effects of the thermogenic adipose tissue on T2D and its complications

The prevalence of T2D is rising at an alarming rate, placing an enormous burden on human health. It has been shown that increasing the metabolic activity of the BAT and beige adipose tissue may be a new way to reduce circulating glucose and blood lipids in patients with T2D and that T2D is characterized by the reduced metabolic activity of the BAT. Therefore, restoring BAT function may be an effective way to treat T2D. Cold exposure and β3-AR agonists activate adenosine 5'-monophosphate (AMP)-activated protein kinase (AMPK), which has been shown to increase the metabolic activity of the human BAT. Researchers have found that the loss of AMPK in adipocytes exacerbates the development of hepatic steatosis, hepatic insulin resistance, and systemic glucose and insulin intolerance 106. Similarly, mice with adipose tissue-specific deletion of the AMPK β1β2 receptor developed more severe systemic insulin resistance. The activation of AMPK can promote WAT browning and prevent HFD-induced obesity and related metabolic dysfunction 107.

Diabetic nephropathy is a progressive decrease in proteinuria and glomerular filtration rate of the kidney, which is caused by long-term diabetes. It is one of the most important comorbidities of patients with diabetes. Complex metabolic disorders once developed into end-stage renal disease are often more problematic than other kidney diseases. Previous studies have shown that BAT can regulate metabolism by releasing cytokines. Also, the sustained stimulation of the BAT contributes to the release of adipokines, which can have beneficial effects on the kidneys 108-111. In addition, the AMPK signaling pathway can also play a positive role in the diabetic kidney, such as the activation of the AMPK/Sirt1/PGC1α axis to prevent inflammation and oxidative stress by inhibiting the NF-κB pathway and promoting fatty acid β-oxidation and the expression of antioxidants 112-114. Cai *et al.* found that the expressions of BAT-specific genes and fatty acid metabolism-related genes were down-regulated in diabetic mice, which were restored after treatment with the β3-AR agonist CL316,243. Also, the activation of the BAT enhanced its sensitivity to FGF21, and FGF21 can activate the AMPK signaling pathway. In addition, CL316,243 treatment activates the AMPK/Sirt1/PGC1α axis in diabetic kidneys, which suggests that CL316,243 treatment can play an anti-oxidative and anti-inflammatory role in diabetic kidneys 115.

### Effects of thermogenic adipose tissue on other metabolic-related diseases

**Figure 5:**
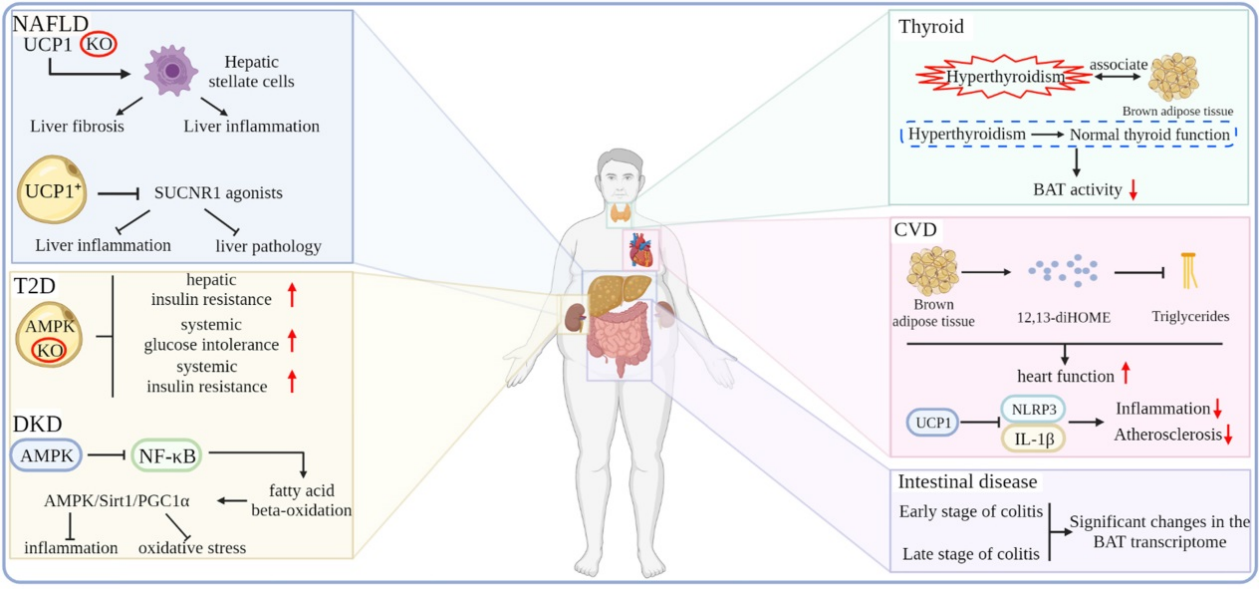
Effects of the thermogenic adipose tissue on obesity-related metabolic diseases.

In recent years, CVD has become a major factor that endangers human health. CVD includes conditions, such as arrhythmias, heart failure, and atherosclerosis, which can cause fatal injuries, such as myocardial infarction and cardiac arrest. CVD can be caused by a variety of factors, including obesity, which increases the risk of CVD by increasing the development of dyslipidemia and diabetes. The BAT can affect the metabolism of the whole body in the form of endocrine. Studies have shown that the BAT under cold exposure conditions increases the uptake of fatty acids by the BAT and skeletal muscle in an autocrine or paracrine manner by releasing 12,13-diHOME. Subsequent studies have shown that BAT is a direct endocrine function that enhances cardiac function by regulating calcium cycling through 12,13-diHOME and NOS1 116-118. Atherosclerosis is a chronic inflammatory disease formed by fatty acid deposits in the innermost layer of arteries and is a major cause of CVD. Obesity-related adipose dysfunction is among the main culprits of atherosclerosis. Studies have shown that the BAT marker gene UCP1 exerts anti-inflammatory and anti-atherosclerotic effects by blocking mitochondrial superoxide-induced nod-like receptor family pyrin domain 3 inflammasome activation and interleukin-1β production 119. In addition, BAT also plays a role in regulating intestinal disease tolerance. The BAT transcriptome is dramatically altered in the early and late stages of dextran-sodium sulfate (DSS)-induced colitis in mice without inhibiting the oxygen consumption rate under hypothermic conditions, thus suggesting that BAT responds dynamically to DSS-induced colonic injury and demonstrated the existence of a signaling axis between thermogenic adipocytes and intestinal epithelial cells, which regulates disease tolerance 120. One study performed nuclear imaging of the BAT activity coupled in subjects and followed the carotid artery anatomy and functional vascular imaging for up to five years, demonstrating that an increased BAT activity may potentially reduce the incidence of subclinical atherosclerosis and improve vascular function rather than increase the risk of CVD 121. In addition to the diseases closely related to obesity, other diseases are related to BAT, such as hyperthyroidism. Studies have shown that hyperthyroidism is related to activate BAT. With normal thyroid function, the activity of the BAT gradually decreases 122 **(Figure 5)**.

## Conclusion

Obesity is one of the major factors in many metabolic disorders and there are a growing number of treatments for obesity and its metabolic syndrome, among which increased energy expenditure through activation and recruitment of brown or beige adipocytes is effective for the treatment of obesity. The roles of cRNAs and ncRNAs in obesity and metabolic syndrome have been widely reported; however, studies on their roles in adipose tissue thermogenesis are still at the tip of the iceberg. This article reviews the research progress of cRNAs regulating adipose tissue thermogenesis in several widely studied pathways by regulating typical thermogenesis key factors and miRNAs/lncRNAs in ncRNAs. This article also reviews the role of thermogenic adipose tissue in regulating glycolipid metabolism. Relevant advances in this field have deepened our understanding of the regulation of thermogenesis in thermogenic adipose tissue and provided new strategies and approaches for promoting energy expenditure and treating obesity and its metabolic syndrome.

## Outlook

Nowadays, there are several scientific research methods and several technologies are being developed. Some emerging research methods can be introduced into the study of adipose tissue, such as the three-dimensional genome of adipocytes and single-cell multi-omics sequencing, to discover more functional genes that play a role in the function of adipose tissue 123-125. In addition, in recent years, tRNA-derived RNA fragments and piRNAs in ncRNAs have also attracted the attention of researchers 126, 127, and their roles in adipose tissue are still worthy of our attention and exploration.

## Figures and Tables

**Table 1:** miRNA/lncRNA targets non-classical thermogenesis factors to regulate adipose tissue thermogenesis.

Name	Mechanism	Function	References
miR-22	Target and inhibit Tsc1	Impairs glycolytic capacity in BAT, which is critical for thermogenesis.	76
miR-337-3p	Target and inhibit* Twist1*	Increase browning marker gene expression and promote adipocyte browning	77
miR-33	Target and inhibit Zfp516 and Dio2	Inhibition of miR-33 upregulates the expression of target genes and UCP1 in brown and subcutaneous white adipose tissue and increases the cold resistance of mice.	78
LncRNA Blnc1	Forms a ribonucleoprotein complex with hnRNPU and EBF2	Regulate thermogenic gene transcription and promote thermogenic adipocyte differentiation	79
LncRNA AK079912	Adjustment by PPARγ	Promotes brown adipocyte production and white adipose tissue browning.	80

## Abbreviations

T2D: type 2 diabetes
cRNA: coding RNA
ncRNA: non-coding RNA
miRNA: microRNA
lncRNA: long noncoding RNA
WAT: white adipose tissue
WHO: the World Health Organization
BAT: brown adipose tissue
UCP1: uncoupling protein 1
ETC: electron transport chain
β3-AR: β3-adrenergic receptor
mRNA: messenger RNA
rRNA: ribosomal RNA
tRNA: transfer RNA
snRNA: small nuclear RNA
piRNA: Piwi-interactingRNA
NE: norepinephrine
cAMP: cyclic adenosine monophosphate
CREB: cAMP-responsive element-binding protein
HFD: high-fat diet
DRP1: dynamin-related protein 1
mTOR: The mammalian target of rapamycin
CVD: cardiovascular disease
NAFLD: non-alcoholic fatty liver disease
HSC: Hepatic stellate cell
NASH: nonalcoholic steatohepatitis
AMPK: adenosine 5'-monophosphate (AMP)-activated protein kinase
DSS: dextran-sodium sulfate
